# Characterizing population-wide genomic risk distribution for development of a novel clinical-genomic risk system for prognostication in patients with clinically localized prostate cancer

**DOI:** 10.1038/s41391-025-01062-8

**Published:** 2025-12-09

**Authors:** Udit Singhal, Ralph Jiang, James A. Proudfoot, Elizabeth Chase, Krithika Suresh, Elai Davicioni, Tudor Borza, Michael J. Zelefsky, Brian J. Davis, Brad J. Stish, R. Jeffrey Karnes, Stephen J. Freedland, Martha K. Terris, William J. Aronson, Matthew R. Cooperberg, Fabio Y. Moraes, Alejandro Berlin, Curtiland Deville, Nicholas G. Zaorsky, Soumyajit Roy, Angela Y. Jia, Jonathan E. Shoag, William C. Jackson, Daniel E. Spratt, Matthew Schipper, Todd M. Morgan, Robert T. Dess

**Affiliations:** 1 Department of Urology, Rogel Cancer Center, University of Michigan, Ann Arbor, MI 48109 USA; 2 Department of Radiation Oncology, University of Michigan, Ann Arbor, MI 48109 USA; 3 Department of Biostatistics, University of Michigan, Ann Arbor, MI 48109 USA; 4 Veracyte Inc., San Francisco, California, 94080 USA; 5 Department of Radiation Oncology, NYU Grossman School of Medicine, New York, NY 10016 USA; 6 Department of Radiation Oncology, Mayo Clinic, Rochester, MN 55905 USA; 7 Department of Urology, Mayo Clinic, Rochester, MN 55905 USA; 8 Department of Urology, Samuel Oschin Comprehensive Cancer Institute, Cedars-Sinai Medical Center, Los Angeles, CA 90048 USA; 9 Section of Urology, Durham VA Medical Center, Durham, NC 277710 USA; 10 Department of Urology, Medical College of Georgia at Augusta, Augusta, GA 30912 USA; 11 Department of Urology, University of California Los Angeles Medical Center, Los Angeles, CA 90095 USA; 12 Department of Urology, University of California San Francisco Medical Center, San Francisco, CA 94158 USA; 13 Department of Oncology, Kingston General Hospital, Queen’s University, Kingston, ON Canada; 14 Department of Radiation Oncology, University of Toronto, Toronto, ON Canada; 15 Department of Radiation Oncology and Molecular Radiation Sciences, Johns Hopkins University, Baltimore, MD 21287 USA; 16 Department of Radiation Oncology, University Hospitals Seidman Cancer Center, Case Western Reserve University School of Medicine, Cleveland, OH 44106 USA; 17 Department of Radiation Urology, University Hospitals Seidman Cancer Center, Case Western Reserve University School of Medicine, Cleveland, OH 44106 USA

**Keywords:** Prostate cancer, Cancer genetics

## Abstract

**Purpose:**

Genomic classifiers are endorsed by guidelines and commonly used to inform prognosis in prostate cancer. We aimed to understand the distribution of genomic risk within the validated staging collaboration for cancer of the prostate (STAR-CAP) and propose a system integrating genomic and clinicopathologic risk. We hypothesized that genomic heterogeneity would have implications on risk estimates and may inform treatment decisions.

**Materials and methods:**

Genomic risk was assessed using the Decipher genomic classifier in two separate multi-institutional, prospectively collected population-based registries: (1) Decipher Genomics Resource for Intelligent Discovery (GRID) [*n* = 50,891] and (2) Michigan Urological Surgery Improvement Collaborative (MUSIC-Decipher) [*n* = 1602]. The primary endpoint was estimated prostate cancer-specific mortality (PCSM), and secondary endpoint was distant metastasis (DM). Marginal risk estimates provided by STAR-CAP were combined with hazard ratios of Decipher to calculate integrated risk estimates.

**Results:**

Median age and PSA was 68 years and 6.2 ng/mL in GRID, and 66 years and 10.5 ng/mL in MUSIC. The GRID population had 50.2%, 18.5%, and 31.4% with low-, intermediate-, and high-Decipher risk, compared to 48.0%, 16.2%, and 35.8% in MUSIC. Decipher-based genomic risk varied across STAR-CAP stages in both registries. Estimates of 10-year PCSM (0.1% to 48.8%) and DM (0.3%-72.9%) varied widely after integration of clinical-genomic risk. Use of an integrated Decipher-STAR-CAP system led to significant stage reclassification, including 23.4% upstaging and 45.6% downstaging at least one stage.

**Conclusions:**

These findings suggest integration of genomic and clinicopathologic risk may lead to more nuanced risk assessment in prostate cancer and may help individualize treatment consideration.

## Plain language summary

Prostate cancer risk assessment traditionally involves consideration of clinical, pathologic, and genetic factors separately. Combining these factors into single risk assessment system may provide more accurate prediction of outcomes, thereby providing more nuanced and individualized risk prediction.

## Introduction

Localized prostate cancer has substantial oncologic heterogeneity, with outcomes ranging from cure to indolent chronicity, progression, and metastasis [1–3]. While accurately predicting risk of disease progression is critical for tailoring treatment approaches, the availability of validated, guideline-endorsed, predictive measures currently available with demonstrated utility in localized disease is sparse. Thus, traditional approaches for risk stratification include consideration of clinical, pathologic, and genomic variables. Pre-treatment stage systems primarily consist of categorical groupings of clinicopathologic factors, including prostate specific antigen (PSA), Gleason score, and clinical T-stage and extent of biopsy involvement [4–8], while genomic risk assessment is primarily based on gene expression analysis from tumor specimens [9]. Despite the availability of multiple such tools for prognostication, models based on clinicopathologic risk assessment have been largely developed in isolation and report separately from those based on prediction of genomic risk, making integration of the different modalities a challenge. Further, variations in genomic risk distribution across patients with similar clinicopathologic stage have been previously demonstrated, representing an additional challenge for large-scale, population-based risk assessment without integrated measures [10]. Additionally, NCCN risk groups represent broad, heterogeneous groups, not fully optimal for contemporary practice. Thus, there remains a need to further refine prognostic classifications and vacate a one-size-fits-all approach for risk stratification. Prior studies have attempted to combine clinical, pathologic, and genomic variables and shown improved ability for predicting outcomes or reclassifying patients with varying clinico-genomic risk [11, 12].

Here, we build upon these prior studies by integrating genomic risk estimation into a previously validated, American Joint Committee on Cancer (AJCC)-compliant clinicopathologic risk system using two large, prospectively collected, population-based registries [8]. We hypothesized that population-based genomic heterogeneity would have implications regarding risk estimates of cancer-related outcomes for patients.

## Materials & methods

### Study population

Genomic risk was assessed using the Decipher genomic classifier (Veracyte Inc.) in two separate multi-institutional, prospectively collected population-based registries: (1) Decipher Genomics Resource for Intelligent Discovery (GRID) [*n* = 50,891] and (2) Michigan Urological Surgery Improvement Collaborative (MUSIC-Decipher) [*n* = 1602]. The Decipher GRID database (NCT02609269) is a prospectively collected resource, including transcriptomic profiles from patients with urologic cancers who have undergone Decipher testing. Those within GRID with biopsy data were included in the current analysis. The MUSIC registry includes data from a statewide collaborative funded by Blue Cross Blue Shield of Michigan, with representation from >90% of urology practices within the state. All practices within MUSIC have exemption or approval by local internal review board for participation within the collaborative. Those who did not undergo Decipher testing within MUSIC were excluded [13].

### Outcome measures

Estimates of genomic risk by Decipher were integrated into a previously validated, AJCC-compliant clinical prognostic group staging system, the staging collaboration for cancer of the prostate (STAR-CAP) [8]. The primary endpoint was prostate cancer-specific mortality (PCSM) with a secondary endpoint of distant metastasis (DM), with estimates of DM and PCSM based upon the STAR-CAP database. Marginal risk estimates provided by STAR-CAP stage were combined with hazard ratios of Decipher on PCSM and DM along with the distribution of Decipher scores by STAR-CAP stage to calculate integrated outcome estimates.

### Statistical analysis

Decipher was modeled as a 10-level ordinal variable (0-.09, .10-.19, … .9-1.0). The goal of the statistical analysis was to estimate the risk of PCSM and DM at 10 years as a function of STAR-CAP (9 level ordinal variable) and Decipher. The available inputs consisted of the distribution of Decipher by STAR-CAP in the GRID and MUSIC-Decipher datasets, 10-year PCSM and DM risk for each STAR-CAP stage [8], and the hazard ratio (HR) per in nominal change Decipher score from multivariable models adjusting for clinical factors as reported in retrospective analyses of prospective randomized trials [14, 15]. Specifically, the Decipher HRs used for PCSM were calculated from the published values of 1.23 (per increase in Decipher of .10) in a high-risk cohort^16^ and 1.45 (per increase in Decipher of 0.10) in a intermediate-risk cohort [17]. For DM, published values of 1.22 and 1.28 were used in the high-risk and intermediate-risk cohorts, respectively [16, 17]. For each STAR-CAP stage, we calculated the Decipher-incorporated risk estimates by constraining the weighted average of risks across the 10 Decipher categories (in 0.1 increments) to equal the marginal risk estimate based on STAR-CAP stage. Moreover, within each STAR-CAP stage, nominal Decipher changes (per 0.1) were set to be consistent with the published Decipher hazard ratios on PCSM or DM for that STAR-CAP stage. The weights were calculated in the MUSIC-Decipher and GRID datasets as the proportion of patients in each Decipher category for a particular STAR-CAP stage. Combining these two constraints determines a unique set of risk estimates. This process was repeated for each STAR-CAP stage to obtain risk estimates for every combination of STAR-CAP stage and Decipher. Additional details on the statistical methods are provided in the supplementary methods. To calculate a genomically informed STAR-CAP stage, we utilized the Decipher-informed 10-year PCSM risk estimates. Patients were reclassified into the STAR-CAP stage whose marginal (ignoring Decipher) 10-year PCSM risk estimate was closest to the new Decipher-informed 10-year PCSM risk estimate.

### Estimation of the Hazard Ratio (HR) for Decipher

Spratt et. al reported that for intermediate risk patients, the HR for Decipher on PCSM was 1.45 (per 0.1-unit increase) from a multivariable model adjusting for clinical factors. Separately, Nguyen et al., reported that for higher risk patients, the corresponding HR was 1.23. Together these results suggest that, on the HR scale, the effect of Decipher is less (i.e. HR closer to 1.0) for higher STARCAP stage. Thus, we set the Decipher HR as 1.45 in STARCAP IA and 1.23 in STARCAP IIIC. We solved for Decipher HRs in the middle STARCAP stages through logarithmic interpolation. That is, we computed the evenly spaced 9-length sequence from log(1.45) to log(1.23) and exponentiated it to obtain our 9 Decipher HRs. A similar procedure was repeated for DM HRs.

### Sensitivity analysis

Due to concerns about extrapolation, in our main analysis for STAR-CAP IA we set the Decipher hazard ratios for PCSM at 1.45 and DM at 1.28. The corresponding values in STAR-CAP IIIC were 1.23 and 1.22, respectively. We performed a sensitivity analysis where these hazard ratios were instead set for STAR-CAP stages IC and IIIB, to more closely reflect the populations these hazard ratios were estimated from. We used the same method to generate hazard ratios for the other STAR-CAP stages, interpolating between stages IC and IIIB and extrapolating the pattern for stages IA, IB, and IIIC. For PCSM, this resulted in a hazard ratio of 1.55 for stage IA and 1.19 for stage IIIC (Supplementary Fig. 1). For DM, this resulted in a hazard ratio of 1.30 for stage IA and 1.21 for stage IIIC (Supplementary Fig. 2).

## Results

### Baseline characteristics

A total of 52,493 patients were included in the analysis, including 1602 and 50,891 in the MUSIC-Decipher and GRID populations, respectively. Median age and PSA was 66 years and 10.6 ng/mL in MUSIC-Decipher (Table 1) and 68 years and 6.2 ng/mL in GRID (Supplementary Table 1). The most common Gleason Grade Group (GG) was GG2 (41.7% MUSIC-Decipher, 41.4% GRID). Most patients included those with clinical stage T1c disease (76.5% MUSIC-Decipher, 82.8% GRID). The MUSIC-Decipher population included 13% Black patients, compared to 10% in the GRID population. The MUSIC-Decipher population had 48%, 16.2%, and 35.8% with low-, intermediate-, and high-Decipher risk, compared to 50.2%, 18.5%, and 31.4% in GRID.

**Table 1:** Baseline characteristics of the MUSIC and GRID populations.

	Low Decipher (*N* = 769)	Intermediate Decipher (*N* = 260)	High Decipher (*N* = 573)	Overall (*N* = 1602)
**Age**				
Mean (SD)	64.5 (7.90)	66.7 (7.53)	68.1 (8.31)	66.1 (8.15)
Median [Q1, Q3]	65.0 [59.0, 70.0]	68.0 [62.0, 72.0]	69.0 [63.0, 74.0]	66.0 [61.0, 72.0]
**Race**				
Black	92 (12.0%)	41 (15.8%)	76 (13.3%)	209 (13.0%)
Asian	6 (0.8%)	2 (0.8%)	8 (1.4%)	16 (1.0%)
Other	59 (7.7%)	18 (6.9%)	50 (8.7%)	127 (7.9%)
White	595 (77.4%)	196 (75.4%)	431 (75.2%)	1222 (76.3%)
Missing	17 (2.2%)	3 (1.2%)	8 (1.4%)	28 (1.7%)
**PSA**				
Mean (SD)	6.70 (8.79)	13.6 (77.2)	14.4 (42.9)	10.6 (40.9)
Median [Q1, Q3]	5.51 [4.24, 7.37]	5.90 [4.40, 8.71]	6.78 [4.80, 11.1]	5.96 [4.41, 8.50]
**Grade Group**				
1	292 (38.0%)	52 (20.0%)	59 (10.3%)	403 (25.2%)
2	373 (48.5%)	127 (48.8%)	168 (29.3%)	668 (41.7%)
3	89 (11.6%)	63 (24.2%)	194 (33.9%)	346 (21.6%)
4	11 (1.4%)	10 (3.8%)	98 (17.1%)	119 (7.4%)
5	4 (0.5%)	8 (3.1%)	54 (9.4%)	66 (4.1%)
**Stage at Biopsy**				
T1a	7 (0.9%)	0 (0%)	3 (0.5%)	10 (0.6%)
T1b	1 (0.1%)	1 (0.4%)	0 (0%)	2 (0.1%)
T1c	650 (84.5%)	213 (81.9%)	362 (63.2%)	1225 (76.5%)
T2a	71 (9.2%)	28 (10.8%)	96 (16.8%)	195 (12.2%)
T2b	16 (2.1%)	6 (2.3%)	51 (8.9%)	73 (4.6%)
T2c	24 (3.1%)	10 (3.8%)	49 (8.6%)	83 (5.2%)
T3	0 (0%)	1 (0.4%)	11 (1.9%)	12 (0.7%)
T4	0 (0%)	1 (0.4%)	1 (0.2%)	2 (0.1%)
**STARCAP**				
IA	117 (15.2%)	18 (6.9%)	21 (3.7%)	156 (9.7%)
IB	143 (18.6%)	24 (9.2%)	32 (5.6%)	199 (12.4%)
IC	243 (31.6%)	75 (28.8%)	82 (14.3%)	400 (25.0%)
IIA	143 (18.6%)	69 (26.5%)	133 (23.2%)	345 (21.5%)
IIB	63 (8.2%)	39 (15.0%)	98 (17.1%)	200 (12.5%)
IIC	22 (2.9%)	13 (5.0%)	73 (12.7%)	108 (6.7%)
IIIA	22 (2.9%)	5 (1.9%)	39 (6.8%)	66 (4.1%)
IIIB	13 (1.7%)	16 (6.2%)	67 (11.7%)	96 (6.0%)
IIIC	3 (0.4%)	1 (0.4%)	28 (4.9%)	32 (2.0%)

### Genomic-risk distribution

**Fig. 1:**
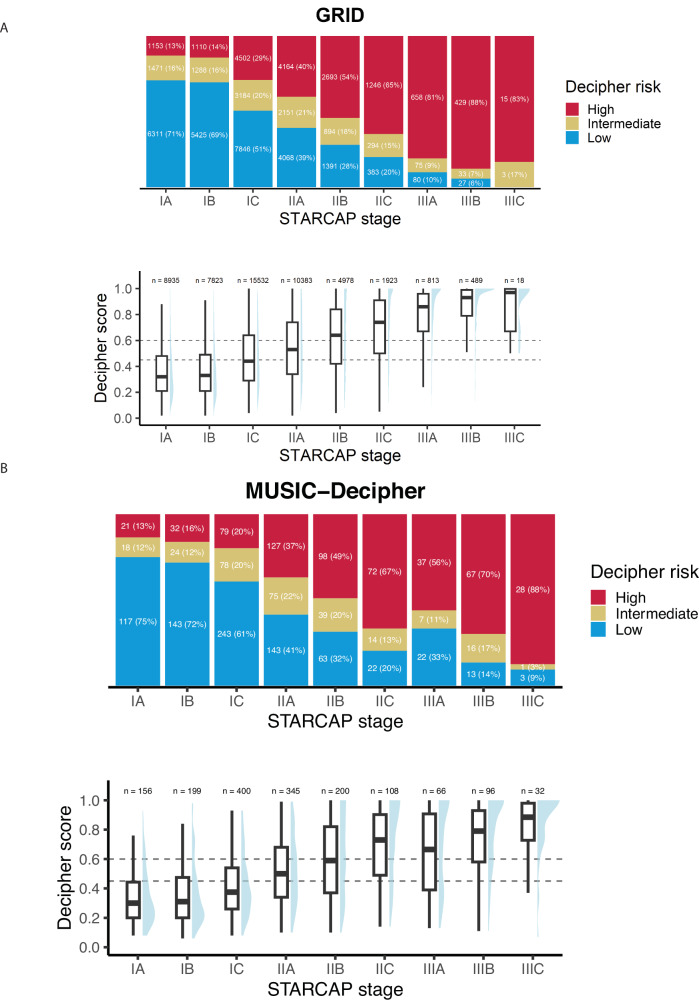
Genomic Risk Distribution in Decipher GRID and MUSIC populations. Genomic risk distribution (by quartile; y-axis) by Decipher genomic classifier amongst AJCC compliant STAR-CAP clinicopathologic stage groups (x-axis) in the **A** Decipher-GRID (*n* = 50,981) and **B** MUSIC-Decipher (*n* = 1584) populations. Dashed lines signify separation between Decipher risk groupings. Density plot represents distribution of patients among the different Decipher risk within the separate STARCAP stages.

Decipher-based genomic risk varied across all STAR-CAP stages (IA-IIIC) in both the GRID (Fig. 1a) and MUSIC-Decipher registries (Fig. 1b). For example, among those with low STAR-CAP stage (IA-IC), there was a proportion of men with high genomic risk in both the GRID (18.7%) and MUSIC-Decipher (17.9%) populations. Conversely, in those with the highest risk by clinicopathologic assessment (STAR-CAP IIIA-IIIC), there was a subset of patients with low genomic risk, including 15.3% in GRID and 19.6% in MUSIC-Decipher, suggesting substantial variability within and across clinicopathologic groupings.

### Outcome assessment and reclassification

**Fig. 2:**
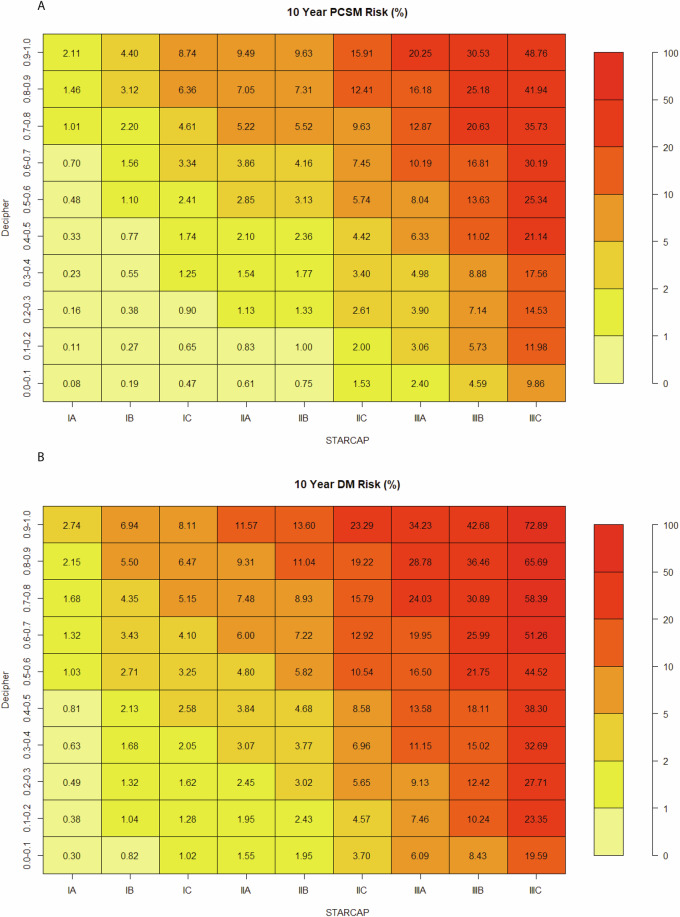
Heatmap representation of Decipher-STARCAP risk estimation. Heatmap of estimated percentage of 10-year (**A**) PCSM and (**B**) DM according to Decipher risk decile (y-axis) and STAR-CAP stage (x-axis).

**Fig. 3:**
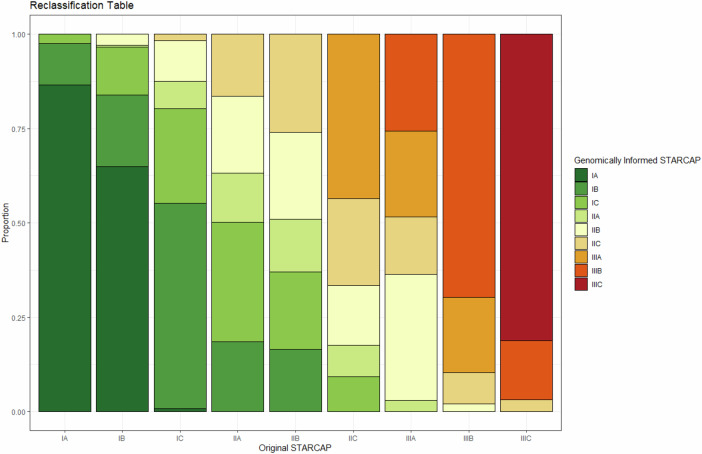
Reclassification assessment according to genomically-informed STAR-CAP (color-coded), compared to original STAR-CAP stage (x-axis).

Integrated estimates of genomic (by Decipher) and clinicopathologic risk (by STAR-CAP) were calculated as 10-year PCSM and DM (Fig. 2) across all staging combinations. Risk of 10-year PCSM ranged from <1% (STAR-CAP IA and Decipher 0.0-1) to 48.8% (STAR-CAP IIIC and Decipher 0.9-1.0). Within each STAR-CAP stage, predicted PCSM risk varied substantially depending on Decipher score (Supplementary Table 2). For example, for a STAR-CAP IIA patient with a high Decipher score (0.86 = the 90^th^ percentile for IIA patients) of 10-year PCSM risk was 7.1%, while the PCSM risk for a STAR-CAP IIIA patient with low Decipher score (0.27 = the 10^th^ percentile) was 3.9%. Similarly, estimated 10-year risk of DM ranged from <1% (STAR-CAP IA and Decipher 0.0-1) to 72.9% (STAR-CAP IIIC and Decipher 0.9-1.0). Within each STAR-CAP stage, estimated DM risk varied substantially when Decipher-based genomic risk estimation was added. Use of a Decipher-informed STAR-CAP system led to a significant degree of stage reclassification based on predicted risk of 10-year PCSM (Fig. 3), including 23.4% upstaging at least one stage and 45.6% downstaging at least one stage and 7.4% and 12.2% upstaging or downstaging at least two stages, respectively.

## Discussion

In this study, we assessed population-wide genomic risk distribution and combined genomic and clinicopathologic risk estimation using two large, prospective population-based registries with >50,000 patients to understand if genomic heterogeneity results in variable risk stratification for cancer-related outcomes. We demonstrate the existence of considerable genomic heterogeneity in within similar clinicopathologic risk staging groups, suggesting that even the most granular conventional methods for risk stratification may not fully capture the underlying biological diversity of localized prostate cancer, which has implications for risk stratification. A combined approach based on both genomic and clinicopathologic assessment by Decipher and STAR-CAP, may lead to more nuanced risk estimation of PCSM and DM. For a given patient, for example, with STAR-CAP stage IIC disease, 10-year PCSM risk varied from 1.5–15.9% and 10-year DM risk varied from 3.7-23.3%, depending on the integrated risk assessment. This has significant implications for patient counseling, treatment selection, and determination of suitability for active surveillance in lower risk scenarios or possibility of therapy intensification in higher risk scenarios.

The current study builds upon prior work suggesting improved prognostication using a three- or six-tier combined clinical-genomic risk grouping system using Decipher and NCCN risk groupings [12, 18]. Here, however, we utilize a previously validated, American Joint Committee on Cancer (AJCC)-compliant, staging system with demonstrated ability to better prognosticate cancer-related outcomes compared to existing categorizations, including AJCC, NCCN, and CAPRA [8]. This suggests that despite optimized risk assessment using clinical and pathologic variables, further addition of genomic risk assessment allows for continued additive benefit. This is further supported by the large-scale, population-wide heterogeneity in genomic risk seen in our study, which is consistent with prior investigations suggesting significant biologic inter- and intra-tumoral heterogeneity in prostate cancer [19–22]. For example, in an analysis of biopsy tissue from localized disease, even among those that had homogeneous low-risk disease by traditional clinical characteristics, there was evidence of substantial genomic heterogeneity, with enrichment of PAM50 luminal A, luminal B, and basal subtypes [10]. Combining the CAPRA-S and Decipher genomic classifier resulted in improved prediction of post-prostatectomy cancer-specific mortality amongst the highest risk patients, and allowed for ‘down-staging’ of those with high-risk disease by clinical characteristics found to be low-risk by genomic assessment [11]. In our study, 10-year estimates in all stages for PCSM ranged from 0.1% to 48.8%, and DM from 0.3% to 72.9%. This mirrors the increased discriminatory ability previously reported, where the clinical-genomic risk grouping system exhibited a higher c-index (0.84) compared to NCCN risk groups (0.73), indicating superior accuracy in predicting for the events analyzed; PCSM and DM [12].

Clinically, the integration of genomic data facilitates the potential for more personalized treatment strategies. Patients identified as higher risk through combined assessment may benefit from intensified treatment regimens, while those classified as lower risk could potentially avoid overtreatment and its associated morbidities. Thus, ongoing trials are now evaluating the concept of treatment intensification or de-intensification based on combined clinical-genomic risk assessment (NCT04513717, NCT05050084) and will be crucial in validating the utility of genomic integration into clinical practice. Further evidence for the potential role of genomic classifiers in prostate cancer was demonstrated in a prospective, randomized trial suggesting that genomic classifier testing can impact treatment decision-making in post-prostatectomy patients [23].

Our study is not without limitations. The cohorts utilized, though large and diverse, may not encompass all demographic and geographic variations. Additionally, the integration methodology relies on specific assumptions regarding hazard ratios and risk distributions of Decipher, which may not be universally applicable. For example, Decipher was included linearly so that every increase in 0.1 in Decipher score corresponded to the same relative increase in hazard of DM or PCSM across the range of Decipher. Thus, further validation of this integrated approach will be needed to enhance precision. Lastly, patient outcomes were not available in the datasets utilized, and therefore the modeling was not performed as a direct function of STAR-CAP and Decipher. Nonetheless, we demonstrate potentially additive benefit of combining genomic risk assessment to traditional approaches for risk stratification, despite optimized risk calculation shown to previously outperform other measures. The true benefit of combined risk assessment will be further elucidated with assessment of combined risk performance for predicting outcomes.

## Conclusions

The integration of genomic classifiers like Decipher into clinicopathologic staging systems represents a significant advancement in prognostication. Our findings demonstrate that such integrated systems provide a more nuanced understanding of patient risk, enabling individualized treatment counseling. By bridging the gap between genomic and clinicopathologic assessments, this approach holds promise for individualizing and optimizing care in localized prostate cancer. Future efforts should focus on expanding the applicability of this integrated system to optimize decision-making based upon a combined approach to risk stratification.

## Supplementary information

Supplementary Methods

Supplementary Table 1

Supplementary Table 2

Supplementary Figure 1

Supplementary Figure 2

Supplementary Figure Legends

## Data Availability

The data sets generated during the current study are not publicly available as they are institutional but are available from the corresponding author on reasonable request.

## Code availability

If *R* code is requested for any of the analyses, it will be provided upon reasonable request.
