# Supplementary material for: Characterizing population-wide genomic risk distribution for development of a novel clinical-genomic risk system for prognostication in patients with clinically localized prostate cancer
Source: Prostate Cancer Prostatic Dis. 2025 Dec 9;29(3):570–7. doi: 10.1038/s41391-025-01062-8 (PMC13472942; doi:10.1038/s41391-025-01062-8)

10 Year DM Risk (%), Sensitivity Analysis

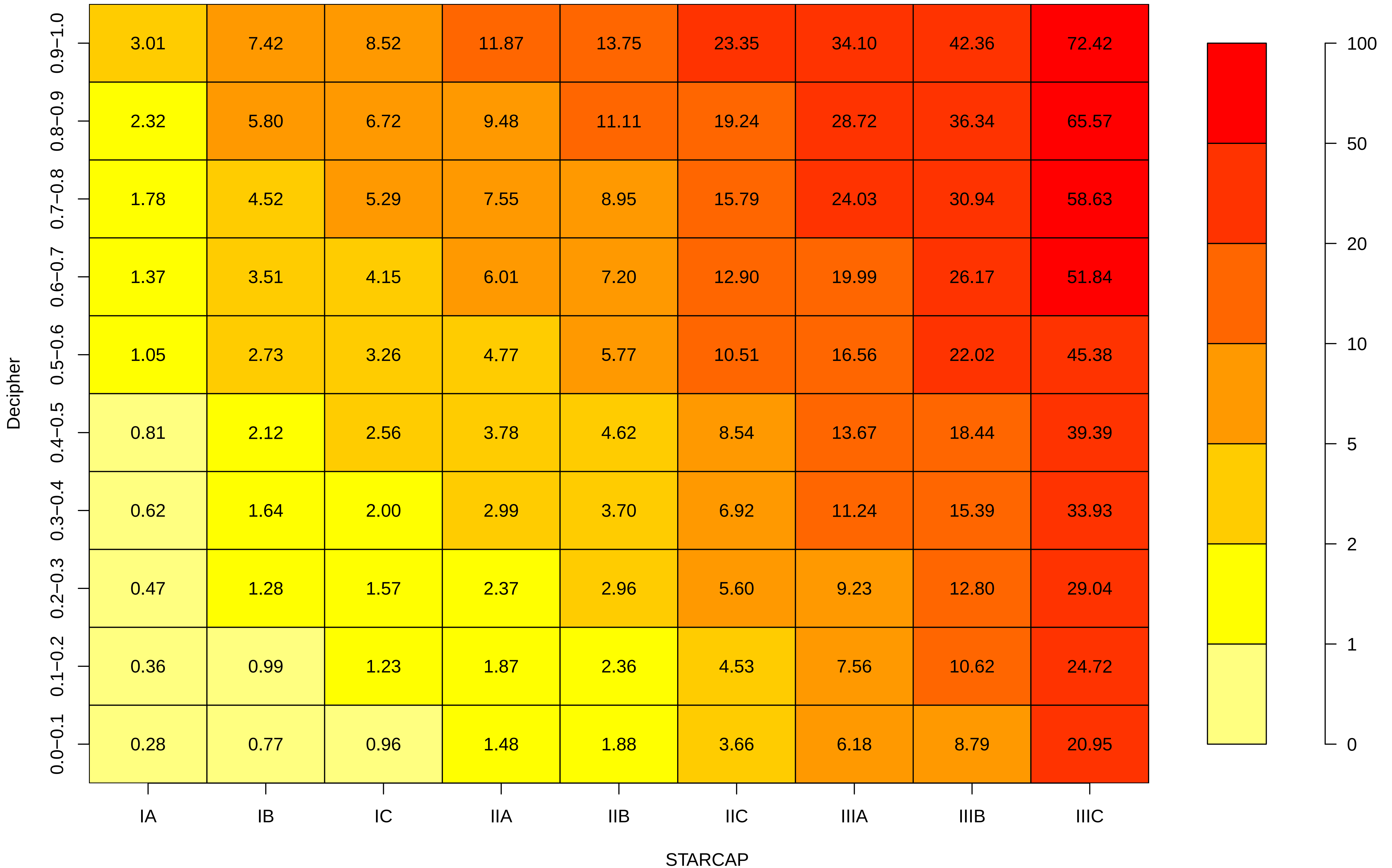

Supplement: Supplementary file 2 [file 41391_2025_1062_MOESM5_ESM.pdf]
