# Supplementary material for: Characterizing population-wide genomic risk distribution for development of a novel clinical-genomic risk system for prognostication in patients with clinically localized prostate cancer
Source: Prostate Cancer Prostatic Dis. 2025 Dec 9;29(3):570–7. doi: 10.1038/s41391-025-01062-8 (PMC13472942; doi:10.1038/s41391-025-01062-8)
Supplement: Supplementary file 3 [file 41391_2025_1062_MOESM1_ESM.docx]

**Supplementary Methods**

***Estimation of PCSM and DM risk using Decipher and STARCAP:*** Let Y denote the outcome of interest (e.g. 10-year PCSM risk), S denote STARCAP stage, and D denote Decipher category (from 1 to 10, with 1 representing 0-0.1, 10 representing 0.9-1.0). To solve for the 10-year PCSM risk given a patient’s STARCAP stage and Decipher category, we solved the following equations:

(1) $P\left( Y | S=s \right)=\sum_{d=1}^{10} P\left( Y | S=s,D=d \right)*P(D=d|S=s)$

(2)

The first equation sets the marginal probability of 10-year PCSM given STARCAP stage equal to a weighted average of 10-year PCSM probabilities given STARCAP and Decipher, where the weights are the proportion of individuals within each Decipher category, given the STARCAP stage. The marginal probabilities $P(Y|S=s)$ have been previously estimated (Dess et al ref) for each STARCAP stage, and the weights $P(D=d|S=s)$ were estimated from the MUSIC and GRID datasets.

The second set of equations uses the Decipher HRs to relate the survival probabilities of individuals with the same STARCAP stage but different Decipher categories. For example, the survival in Decipher category 2 is equal to the survival in Decipher category 1 raised to the power of the Decipher HR for that STARCAP stage.

For a fixed STARCAP stage, solving these equations yields a unique estimated value for $P(Y|S=s,D=d)$ for $d=1,\ldots,10.$ We solved this series of equations separately for each STARCAP stage, then repeated this process for an outcome of 10-year DM. These estimated values of $P(Y|S=s,D=d)$ are the values shown in the Figure 2 heatmaps.
