# Supplementary material for: Characterizing population-wide genomic risk distribution for development of a novel clinical-genomic risk system for prognostication in patients with clinically localized prostate cancer
Source: Prostate Cancer Prostatic Dis. 2025 Dec 9;29(3):570–7. doi: 10.1038/s41391-025-01062-8 (PMC13472942; doi:10.1038/s41391-025-01062-8)
Supplement: Supplementary file 4 [file 41391_2025_1062_MOESM2_ESM.docx]

**Supplementary Table 1**: Patient and clinical characteristics in the prospective GRID cohort by Decipher risk group

|  | **Decipher risk group** | | |  |  |
| --- | --- | --- | --- | --- | --- |
|  | Low | Intermediate | High | Overall | p-value |
| Total | 25531 (50%) | 9393 (18%) | 15970 (31%) | 50894 |  |
| **Age at biopsy**; median (Q1, Q3) | 67 (61, 72) | 68 (63, 73) | 69 (64, 75) | 68 (62, 73) | <0.001 |
| **Physician reported race**; n (%) |  |  |  |  |  |
| Other or unreported | 24794 (97%) | 9155 (97%) | 15612 (98%) | 49561 (97%) | <0.001 |
| Black | 737 (3%) | 238 (3%) | 358 (2%) | 1333 (3%) |  |
| **Race**; n (%) |  |  |  |  |  |
| White | 11773 (46%) | 5003 (53%) | 9730 (61%) | 26506 (52%) | <0.001 |
| Indeterminate | 11185 (44%) | 3396 (36%) | 4753 (30%) | 19334 (38%) |  |
| Black | 2573 (10%) | 994 (11%) | 1487 (9%) | 5054 (10%) |  |
| **PSA (ng/mL)**; median (Q1, Q3) | 6.0 (4.6, 8.1) | 6.3 (4.8, 8.7) | 6.7 (5.0, 9.9) | 6.2 (4.7, 8.7) | <0.001 |
| PSA < 4 | 3512 (14%) | 1122 (12%) | 1790 (11%) | 6424 (13%) | <0.001 |
| 4 ≤ PSA < 10 | 18304 (72%) | 6551 (70%) | 10234 (64%) | 35089 (69%) |  |
| 10 ≤ PSA ≤ 20 | 3231 (13%) | 1463 (16%) | 2846 (18%) | 7540 (15%) |  |
| PSA > 20 | 484 (2%) | 257 (3%) | 1100 (7%) | 1841 (4%) |  |
| **Grade group**; n (%) |  |  |  |  |  |
| 1 | 12772 (50%) | 3033 (32%) | 2520 (16%) | 18325 (36%) | <0.001 |
| 2 | 10015 (39%) | 4369 (47%) | 6668 (42%) | 21052 (41%) |  |
| 3 | 2195 (9%) | 1496 (16%) | 3966 (25%) | 7657 (15%) |  |
| 4 | 429 (2%) | 371 (4%) | 1643 (10%) | 2443 (5%) |  |
| 5 | 120 (<1%) | 124 (1%) | 1173 (7%) | 1417 (3%) |  |
| **Stage at biopsy**; n (%) |  |  |  |  |  |
| T1a | 433 (2%) | 164 (2%) | 208 (1%) | 805 (2%) | <0.001 |
| T1b | 90 (<1%) | 28 (<1%) | 44 (<1%) | 162 (<1%) |  |
| T1c | 21771 (85%) | 7828 (83%) | 12533 (78%) | 42132 (83%) |  |
| T2a | 1959 (8%) | 774 (8%) | 1479 (9%) | 4212 (8%) |  |
| T2b | 536 (2%) | 262 (3%) | 748 (5%) | 1546 (3%) |  |
| T2c | 671 (3%) | 274 (3%) | 694 (4%) | 1639 (3%) |  |
| T3a | 60 (<1%) | 41 (<1%) | 166 (1%) | 267 (1%) |  |
| T3b | 10 (<1%) | 18 (<1%) | 81 (1%) | 109 (<1%) |  |
| T4 | 1 (<1%) | 4 (<1%) | 17 (<1%) | 22 (<1%) |  |
