# Supplementary material for: Characterizing population-wide genomic risk distribution for development of a novel clinical-genomic risk system for prognostication in patients with clinically localized prostate cancer
Source: Prostate Cancer Prostatic Dis. 2025 Dec 9;29(3):570–7. doi: 10.1038/s41391-025-01062-8 (PMC13472942; doi:10.1038/s41391-025-01062-8)
Supplement: Supplementary file 5 [file 41391_2025_1062_MOESM6_ESM.docx]

**Supplementary Figure 1 Legend:**

A sensitivity analysis in which hazard ratios were set for STAR-CAP stages IC and IIIB, to more closely reflect the populations these hazard ratios were estimated from. Heatmap showing hazard ratios for 10-year PCSM risk, from stage IA-stage IIIC.

**Supplementary Figure 2 Legend:**

A sensitivity analysis in which hazard ratios were set for STAR-CAP stages IC and IIIB, to more closely reflect the populations these hazard ratios were estimated from. Heatmap showing hazard ratios for 10-year DM risk, from stage IA-stage IIIC.
